# Early risk assessment for COVID-19 patients from emergency department data using machine learning

**DOI:** 10.1038/s41598-021-83784-y

**Published:** 2021-02-18

**Authors:** Frank S. Heldt, Marcela P. Vizcaychipi, Sophie Peacock, Mattia Cinelli, Lachlan McLachlan, Fernando Andreotti, Stojan Jovanović, Robert Dürichen, Nadezda Lipunova, Robert A. Fletcher, Anne Hancock, Alex McCarthy, Richard A. Pointon, Alexander Brown, James Eaton, Roberto Liddi, Lucy Mackillop, Lionel Tarassenko, Rabia T. Khan

**Affiliations:** 1 Sensyne Health Plc, Schrodinger Building, Heatley Road, Oxford Science Park, Oxford, OX4 4GE UK; 2 Chelsea and Westminster Hospital NHS Foundation Trust, 369 Fulham Road, London, SW10 9NH UK; 3 Academic Department of Anaesthesia and Intensive Care Medicine, Imperial College London, Chelsea and Westminster Campus, 369 Fulham Road, London, SW10 9NH UK; 4 Women’s Centre, Oxford University Hospitals NHS Foundation Trust, John Radcliffe Hospital, Headley Way, Headington, Oxford, OX3 9DU UK; 5 Nuffield Department of Women’s and Reproductive Health, University of Oxford, Women’s Centre, John Radcliffe Hospital, Headley Way, Headington, Oxford, OX3 9DU UK; 6 Institute of Biomedical Engineering, Department of Engineering Science, University of Oxford, Oxford, OX3 7DQ UK

**Keywords:** Diseases, Infectious diseases, Viral infection, Medical research, Translational research, Computational biology and bioinformatics, Machine learning, Prognostic markers, Risk factors

## Abstract

Since its emergence in late 2019, the severe acute respiratory syndrome coronavirus 2 (SARS-CoV-2) has caused a pandemic with more than 55 million reported cases and 1.3 million estimated deaths worldwide. While epidemiological and clinical characteristics of COVID-19 have been reported, risk factors underlying the transition from mild to severe disease among patients remain poorly understood. In this retrospective study, we analysed data of 879 confirmed SARS-CoV-2 positive patients admitted to a two-site NHS Trust hospital in London, England, between January 1st and May 26th, 2020, with a majority of cases occurring in March and April. We extracted anonymised demographic data, physiological clinical variables and laboratory results from electronic healthcare records (EHR) and applied multivariate logistic regression, random forest and extreme gradient boosted trees. To evaluate the potential for early risk assessment, we used data available during patients’ initial presentation at the emergency department (ED) to predict deterioration to one of three clinical endpoints in the remainder of the hospital stay: admission to intensive care, need for invasive mechanical ventilation and in-hospital mortality. Based on the trained models, we extracted the most informative clinical features in determining these patient trajectories. Considering our inclusion criteria, we have identified 129 of 879 (15%) patients that required intensive care, 62 of 878 (7%) patients needing mechanical ventilation, and 193 of 619 (31%) cases of in-hospital mortality. Our models learned successfully from early clinical data and predicted clinical endpoints with high accuracy, the best model achieving area under the receiver operating characteristic (AUC-ROC) scores of 0.76 to 0.87 (F1 scores of 0.42–0.60). Younger patient age was associated with an increased risk of receiving intensive care and ventilation, but lower risk of mortality. Clinical indicators of a patient’s oxygen supply and selected laboratory results, such as blood lactate and creatinine levels, were most predictive of COVID-19 patient trajectories. Among COVID-19 patients machine learning can aid in the early identification of those with a poor prognosis, using EHR data collected during a patient’s first presentation at ED. Patient age and measures of oxygenation status during ED stay are primary indicators of poor patient outcomes.

## Introduction

COVID-19, caused by the severe acute respiratory syndrome coronavirus 2 (SARS-CoV-2), is a novel infectious disease that leads to severe acute respiratory distress in humans. In March 2020, the World Health Organisation declared the outbreak a pandemic and, by November 2020, it had caused more than 55 million confirmed cases and 1.3 million estimated deaths worldwide. Disease severity for COVID-19 varies drastically between patients, including asymptomatic infection, mild upper respiratory tract illness and severe viral pneumonia with acute respiratory distress, respiratory failure and thromboembolic events that can lead to death^1–3^. Initial reports suggest that 6–10% of infected patients are likely to become critically ill, most of whom will require mechanical ventilation and intensive care^2,4^. Currently, few prognostic markers exist to forecast whether a COVID-19 patient may deteriorate to a critical condition and require intensive care. In general, patients can be grouped into three phenotypes, being at risk of thromboembolic disease, respiratory deterioration and cytokine storm^5^. Clinical reports find that age, sex and underlying comorbidities, such as hypertension, cardiovascular disease and diabetes, can adversely affect patient outcomes^6,7^. However, few studies have leveraged machine learning to systematically explore risk factors for poor prognosis and predict patient outcomes from early clinical data.

Increasingly, hospitals collate large amounts of patient data as electronic healthcare records (EHRs). Combined with state-of-the-art machine learning algorithms, these data can help to predict patient outcomes with greater accuracy than traditional methods^8,9^. However, EHR data for COVID-19 remains scarce in the public domain, prompting many authors to focus on statistical analyses instead^2,10–12^. Where machine learning has been applied to COVID-19, results have been promising, but most studies suffer from a lack of statistical power owing to small sample size^13–16^. Jiang et al*.* applied predictive analytics to data from two hospitals in Wenzhou, China, which included 53 hospitalised COVID-19 patients, to predict risk factors for acute respiratory distress syndrome (ARDS)^13^. Exploring the risk factors for in-hospital deaths, Zhou and co-workers used univariate and multivariate logistic regression on data of 191 patients in two hospitals in Wuhan, China^14^. Similarly, Xie et al*.* used logistic regression to predict mortality, training a model on 299 patients and validating it on 145 patients from a different hospital in Wuhan, China^16^. Gong et al*.* used a logistic regression model to identify patients at risk of deterioration to severe COVID-19, applied to the data of 189 patients in Wuhan and Guangdong, China^15^. Studies to date have used a combination of demographics, comorbidities, symptoms, and laboratory tests^13–15,17^. These data typically comprise the patients’ entire historical record, as well as observations collected during the current hospital stay^14,16–18^. While the inclusion of a patient’s full EHR history improves predictive performance, such approaches may be limited in their clinical applicability to early risk-assessment; rarely is the entire EHR of a patient available at the point of presentation in hospital.

In this work, we retrospectively apply machine learning to data of 879 confirmed COVID-19 patients from two tertiary referral urban hospitals in London to predict patients’ risk of deterioration to one of three clinical endpoints: (A) admission to an adult intensive care unit (AICU), (B) need for invasive mechanical ventilation, and (C) in-hospital mortality. We restrict our analysis to EHR data available during a patient’s first presentation in the emergency department (ED) as this more accurately resembles the hospital reality of early-risk assessment and patient-stratification. Our analysis provides a proof of principle for COVID-19 risk assessment, with models achieving a high prediction performance, indicating that patient age, oxygenation status and selected laboratory tests are prime indicators of patient outcome.

## Methods

### Data collection and study design

Anonymised EHR data of patients admitted to a two-site hospital Trust in London, England, between January 1st, 2020 and May 26th, 2020, were gathered by Chelsea & Westminster NHS Foundation Trust (NHS Trust, hereafter). The data was supplied in accordance with internal information governance review, NHS Trust information governance approval, and General Data Protection Regulation (GDPR) procedures outlined under the Strategic Research Agreement (SRA) and relative Data Sharing Agreements (DSAs) signed by the NHS Trust and Sensyne Health plc on 25th July 2018. All analyses were conducted on data with no personal identifying information. Therefore, informed consent was waived by the ethics committee of the Chelsea & Westminster NHS Foundation Trust, which provided ethical approval for the study.

Data encompasses clinical observations collated from inpatient encounters. The analysis was restricted to adult patients aged between 18 and 100 years at the time of their COVID-19 related hospital admission. The latter was defined as an admission with a confirmed SARS-CoV-2 infection determined by quantitative reverse-transcription PCR (qRT-PCR). 63% of patients in the study cohort were male and 37% female (Table 1). The majority were white British (28.3%) or did not state their ethnicity (23.3%) (see also Supplementary Fig. S1).

**Table 1:** Composition of overall patient population.

Demographics	
**Patient age (years)**	
Range	18.0–99.0
Overall mean (std)	67.6 (16.9)
Female mean (std)	70.0 (17.4)
Male mean (std)	66.3 (16.3)
**Sex (number of patients)**	
Female	324 (36.9%)
Male	554 (63.0%)
Unknown	1 (0.1%)
**Ethnicity (number of patients)**	
White British	248 (28.2%)
Not stated	205 (23.3%)
Ethnic other	105 (11.9%)
White other	90 (10.2%)
Asian Indian	65 (7.4%)
Asian other	42 (4.8%)
Unknown	30 (3.4%)
Black African	26 (3.0%)
Black Caribbean	23 (2.6%)
Asian Pakistani	13 (1.5%)
Other	32 (3.6%)

### Cohort definition

A total of 1235 COVID-19 positive patients fell within the observation time and study parameters. From these patients, three cohorts were derived, one for each clinical endpoint, as follows (see also Supplementary Fig. S2). First, patients who did not have information relating to an admission to any hospital department in 2020 were excluded, resulting in 968 patients. Then the following exclusion criteria were applied to each of the considered endpoints: for cohort (A) patients without a documented ward location were excluded; for cohort (B) patients without information on oxygen supply were excluded; for cohort (C) patients without hospital discharge information were excluded. Finally, since our models were trained on data available during a patient’s stay in the ED, we removed patients who did not have a documented ED visit. The final cohorts included 879, 878 and 619 patients for cohorts, A, B and C, respectively (Table 2). No significant differences in composition were found between these three cohorts (Supplementary Table S1).

**Table 2:** Clinical endpoint cohorts.

	Cohort A (AICU admission)	Cohort B (ventilation)	Cohort C (mortality)
Number of patients	879	878	619
Target patients	129 (15%)	62 (7%)	193 (31%)
Control patients	750 (85%)	816 (93%)	426 (69%)

Each cohort was divided into target and control groups (see Table 2). For AICU admission, target patients comprise those that were admitted to an AICU at any time during their hospital stay, while control patients are those that remained in any other ward for their entire admission. Target patients in the ventilation cohort were defined as requiring invasive mechanical ventilation, whereas control patients required no or only minimal breathing assistance. Both categories are based on clinical records of oxygen supply according to Table 3. Note that from hospital data the total number of mechanically ventilated patients was 135. However only 62 of these 135 patients were visible in our analysis. This results from staggered deployment of EHR data in the two hospitals such that one site is understood to lack certain data related to mechanical ventilation. Mortality data was based on the discharge destination (mortuary) in clinical records. All regularly discharged patients were considered part of the control cohort.

**Table 3:** Target and control definition for ventilation cohort.

Category	Clinical observation value
Control	Room air, air/none, nasal cannulae, high flow nasal cannulae, venturi mask, face mask, non-rebreather mask, simple face mask, swedish nose with, oxygen, mask, HFOV, face/tracheostomy mask, CPAP, BiPAP
Target	Ventilator, tracheostomy, CMV, VC-CMV, t-piece, HELIOX, IPPV, SIMV, PC-BIPAP, APRV, CPAP / ASB_SPN / CPAP/PS

### Data processing and feature generation

The data set covered patients’ entire encounter history from presentation at the hospital’s ED to discharge, with a median length of in-hospital stay of 7.2 days. Features were only extracted from data available during a patient’s ED stay (median length of stay of 4.7 h). Variables with less than 5% coverage in the patient population were removed from our analysis resulting in a total of 64 clinical features, including patient demographics (3 in total), vital signs (4 in total), laboratory measurements and clinical observations (57 in total). These features and their coverage across all three cohorts are listed in Supplementary S2. Categorical variables such as patient sex and ethnicity were one-hot encoded. For continuous variables patients may have received multiple test results during their stay. These values were aggregated for each feature to only retain the respective minimum, maximum, mean and last observation value in ED. Missing values in the feature set were imputed with the mean of the training data. For the logistic regression model (see below) each feature was also standardised to zero mean and unit variance based on the training set. To account for the class imbalance in the data set (see Table 2), we applied minority class oversampling to the training data using SMOTE^19^. All models were trained using all features described in Supplementary Table S2. Mean feature values across each of the three cohorts separated by control and target patients are provided in Supplementary Tables S2-S4.

### Patient outcome prediction

Three machine-learning algorithms were benchmarked to predict patient outcomes from EHR data: logistic regression, random forest and Extreme Gradient Boosted Trees (XGBoost). Logistic regression, which predicts the probability of a clinical endpoint as a linear function of the feature space, was used as a baseline algorithm. The model was regularised with elastic net using equal weighting given to L_1_ and L_2_ penalties in order to account for the high dimensionality of the data set relative to the number of observations^20^. A random forest^21^ was trained using 100 trees and splits were evaluated using Gini impurity. Classes were inversely weighted to account for the class imbalance present in the data set. An XGBoost algorithm^22^ was trained with its hyperparameters set to 100 trees, max tree-depth of 6, step-shrinkage of 0.3, no subsampling and L_2_ regularisation, to minimize log-loss. This tree-based algorithm trains decision trees sequentially, with each new tree being trained on the residuals of previous trees.

### Performance evaluation

All methods were evaluated using a threefold cross-validation strategy with a stratified validation split based on target patients. The stratified cross-validation splits were kept the same for all methods. For each cross-validation split an independent model was trained, resulting in three independent models for each method. Results are reported as mean and standard deviation across these independent models. Predictive performance was measured in terms of area under curve (AUC) of the receiver operating characteristic (ROC) and, given the presence of class-imbalance, precision-recall curves are provided to assess expected real-world performance relative to random classifiers. To further account for class imbalance, F1 scores and confusion matrices at each model’s ideal classification threshold as derived by Youden’s J statistic on the ROC curve were also computed.

In order to extract the clinical features most relevant to predictions, permutation feature importance (PFI) was calculated for each model post-hoc^21,23^. To this end, each feature was individually randomised using ten trials per feature. The model’s average precision on the validation sets was then compared to the average precision before the feature had been randomised. The changes in precision were normalised by the sum of absolute changes over all features. Averages and standard deviations over the validation sets from three cross-validation folds have been reported. From these results, statistical significance was assessed by computing *p* values based on a one-sided t-test with the null hypothesis of no significant differences from zero mean.

Accumulated local effects (ALE) were computed to determine the directionality of a feature’s effect on model predictions^24^. Specifically, the feature space was divided into ten percentile bins and each feature’s effect was calculated as the difference in predicted risk between the upper and lower bounds of each bin, leaving all other features unchanged. Binning features in this way can reduce the influence of correlated features often encountered when trying to isolate the effect of a single feature^24^.

### Computation

The entire analysis was carried out in Python 3.6.8 on a Linux-based system. Data were processed using numpy 1.18.1^25^ and pandas 1.0.1^26^. Models were constructed with scikit-learn 0.23.1^27^ and xgboost 1.0.1^22^. PFI was implemented using scikit-learn’s permutation_importance function. Results were visualised in matplotlib 3.1.3^28^ and seaborn 0.10.0.

## Results

### Patient pathways

**Figure 1:**
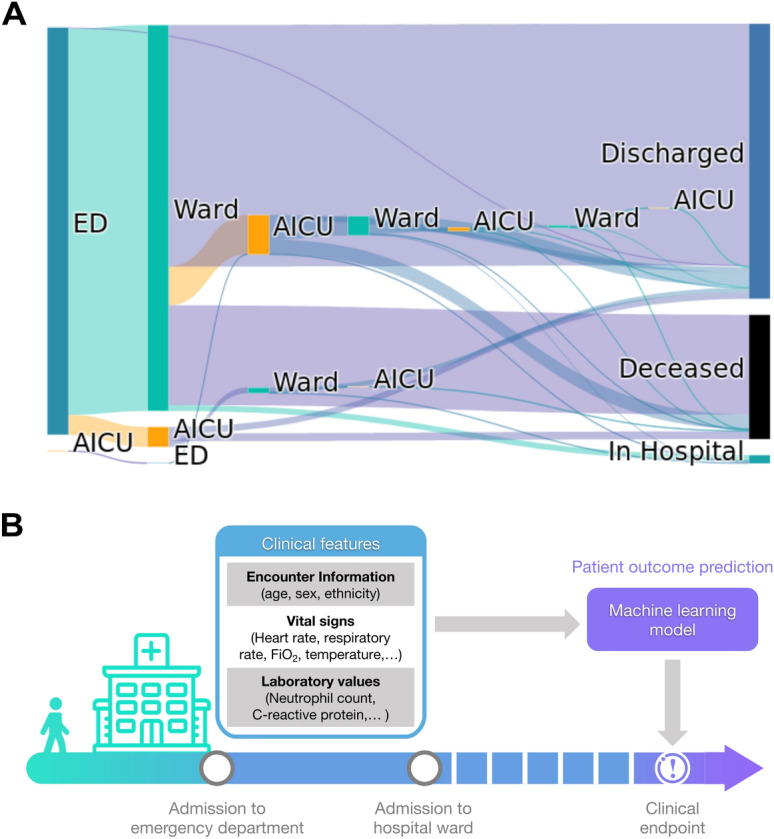
Patient pathways and outcome prediction. (**A**) Patient transitions between hospital departments are shown as bands proportional in size to patient numbers. Different departments are indicted by rectangles (ED, emergency department; Ward, regular hospital ward; AICU, adult intensive care unit). Patients who remain in hospital, are being discharged or die in hospital are indicated on the right. (**B**) Patient outcome prediction models use clinical data recorded within the ED stay of a patient to predict clinical endpoints during the remainder of the in-hospital stay.

A summary of observed patient in-hospital pathways is shown in Fig. 1A. Of the 630 patients for whom the complete pathway is available, 629 (99.8%) entered the hospital via the ED, while 1 (0.2%) patient was admitted directly to AICU. Upon leaving the ED, 596 (94.6%) patients transitioned to regular wards and 32 (5.1%) to an AICU. Of the 596 patients admitted to regular wards, 405 (68%) were ultimately discharged, 12 (2%) remained in hospital and 179 (30%) succumbed to the infection. Among the 596 ward patients, 60 (10.1%) patients required subsequent admission to an AICU. Hence, a total of 92 patients have been admitted to an AICU at some point during their hospital journey. Of these 92 AICU patients, 50 (54.3%) were ultimately discharged, 38 (41.3%) did not survive and 4 (4.3%) remained in hospital.

The median time from hospital presentation to receiving a positive COVID-19 test result was 26.2 h. Patients’ median length of stay in ED was 4.7 h (IQR 3.13 h). During this time, demographic information, vitals and laboratory values were collected (Fig. 1B). To aid an early patient stratification, our models use data collected during the ED stay only to predict whether a patient reached any of three clinical endpoints during their subsequent admission.

### AICU admission

**Figure 2:**
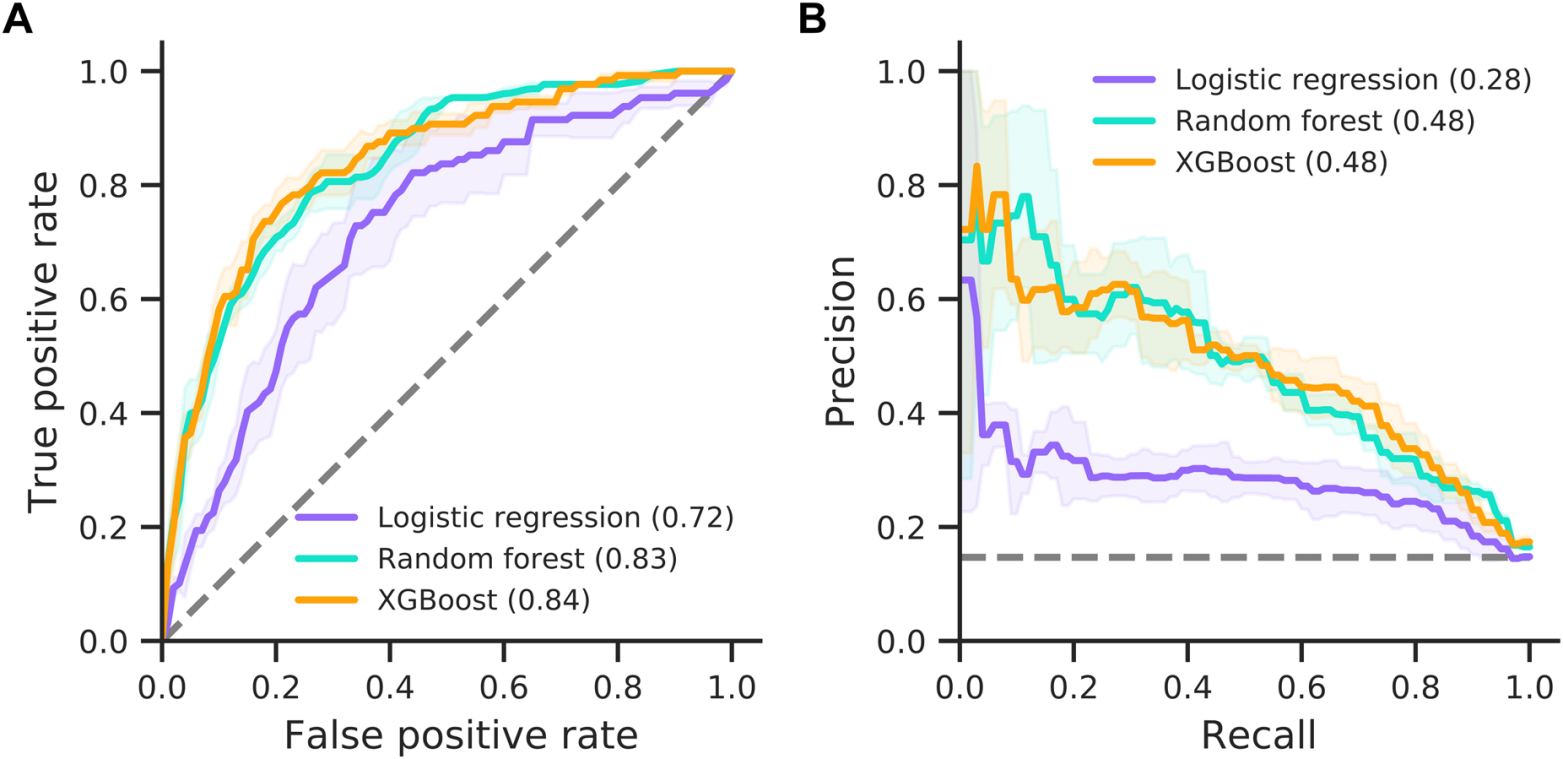
Prediction performance for AICU admission. Model performances for the logistic regression, random forest and XGBoost models are shown as ROC (A) and precision-recall curves (B). AUC is provided in brackets. Solid lines and shaded areas indicate the mean and standard deviation across three cross-validation folds, respectively. Dashed lines indicate random classifiers.

First, we studied patients transitioning to critical care and requiring admission to an AICU. All three models reach good prediction performance on this endpoint, as measured by area under the curve (AUC) of the receiver operating characteristic (ROC) and precision-recall curves, significantly outperforming random classifiers (Fig. 2). The best performing model, XGBoost, reaches an AUC-ROC of 0.84 and an F1 score of 0.52. Both tree-based methods perform better than logistic regression (Table 4). This is to be expected since logistic regression cannot model interactions between features unless such interactions are explicitly encoded into the training data set through feature engineering. To further corroborate model performance on our imbalanced data set (see Table 2), we analysed the calibration of the models’ predicted patient risk against true patient outcomes. Both the random forest and XGBoost models show close to ideal calibration with Brier scores of 0.10, whereas logistic regression is calibrated noticeably worse, reaching a Brier score of 0.21 (Supplementary Fig. S3A). Similarly, both tree-based methods yield a lower number of false positive predictions compared to logistic regression (Supplementary Fig. S4) as well as a higher sensitivity and specificity (Supplementary Table S6). All models show a moderate amount of variability across cross-validation folds (notice standard deviations in Fig. 2 and Table 4), which can compromise subsequent analyses. This instability originates from the limited number of patients and imbalance between target and control patients (see Table 2). Specifically, in each of the three cross-validation folds the models are only trained and validated on two thirds and one third of the data set, respectively, leaving few target patients for these tasks.

**Table 4:** Model performance on clinical endpoint prediction (standard deviation shown in brackets).

Model	Endpoint A (AICU admission)		Endpoint B (ventilation)		Endpoint C (mortality)	
	AUC	F1	AUC	F1	AUC	F1
Logistic regression	0.72 (0.049)	0.40 (0.025)	0.74 (0.082)	0.23 (0.014)	0.70 (0.031)	0.56 (0.028)
Random forest	0.83 (0.005)	0.49 (0.036)	**0.87** (0.028)	0.31 (0.030)	**0.77** (0.030)	**0.61** (0.026)
XGBoost	**0.84** (0.014)	**0.52** (0.012)	**0.87** (0.028)	**0.42** (0.059)	0.76 (0.034)	0.60 (0.032)

Best model in bold.

**Figure 3:**
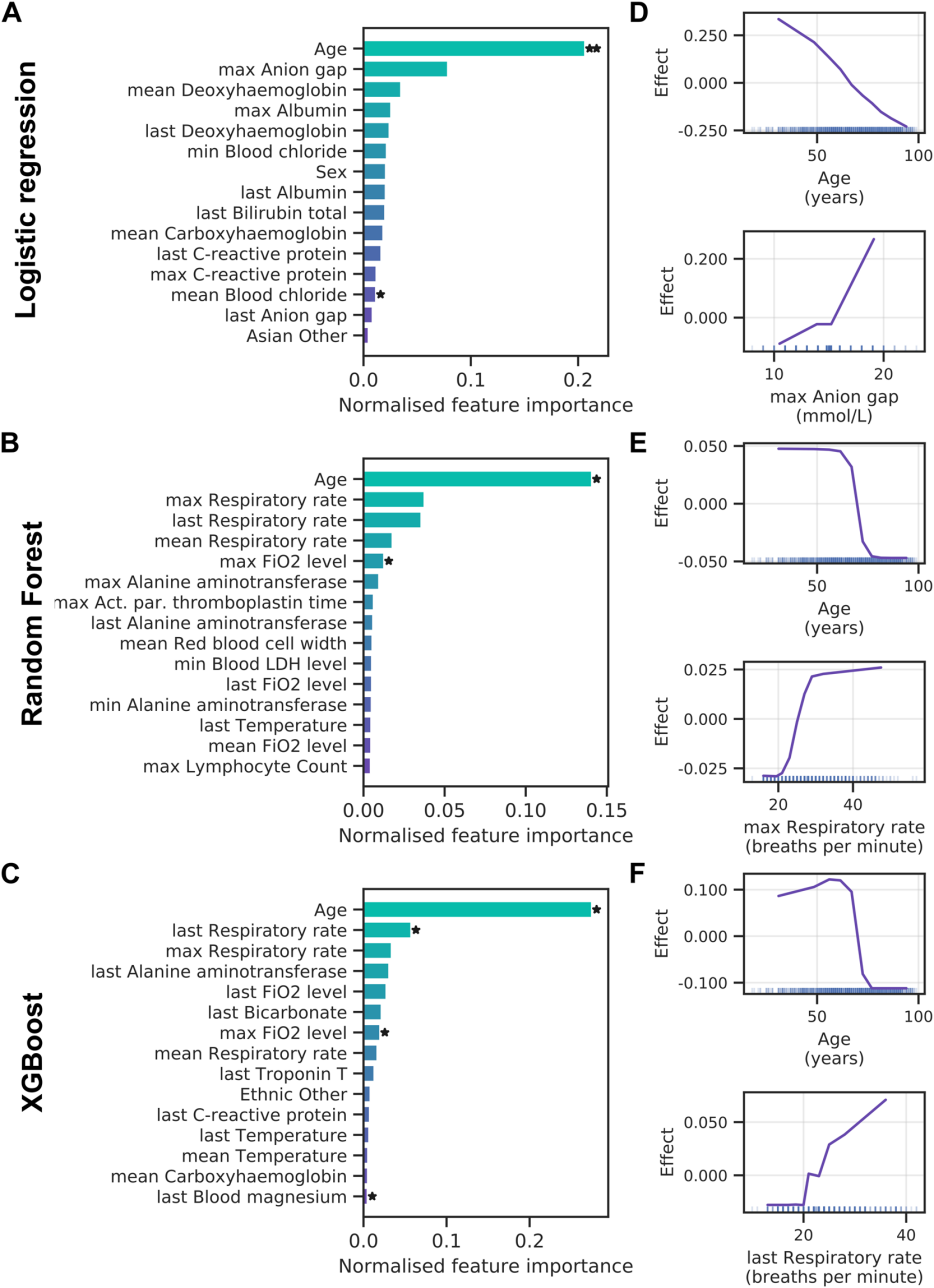
Feature importance for AICU admission. (**A–C**) Permutation feature importance for the logistic regression (**A**), random forest (**B**) and XGBoost (**C**) models. Only the top 15 features are shown. Asterisks mark features with importance scores significantly different from zero across three cross-validation folds with t-test *p* value thresholds of 5% ( ∗) and 1% (∗ ∗). (**D–F**) Accumulated local effects plots for the logistic regression (**D**), random forest (**E**) and XGBoost models (**F**). The top two features according to permutation feature importance are shown for each model. Vertical bars at the bottom indicate feature values observed in the data set.

Next, we assessed which clinical variables contribute the most to model predictions by applying PFI. Figure 3A presents the 15 most important features for the logistic regression with elastic net regularisation. Note that clinical variables that can be recorded multiple times during a patient’s ED visit were aggregated to retain only the minimum, maximum, mean and last observation value during the ED stay. Only patient age reached high importance and significance over cross-validation folds for the logistic regression. The random forest (Fig. 3B) and XGBoost (Fig. 3C) models assign high importance to patient age, with respiratory rate following thereafter. Intriguingly, ALE analyses reveal that lower patient age increases the likelihood of AICU admission in all three models (Fig. 3D–F). This agrees well with a bias towards younger patients when comparing AICU-admitted patients with control patients (Supplementary Fig. S5A). However, clinical indicators of disease severity, such as C-reactive protein and ferritin levels, show no clear trend across age groups (Supplementary Fig. S6). We also find that the anion gap (Fig. 3D) and respiratory rate (Fig. 3E,F) exhibit a positive effect on AICU admission probability.

In summary, machine learning algorithms can predict those COVID-19 patients most likely to require AICU admission from EHR data available during the initial ED stay with high precision. Patient age and measures of oxygenation status are strong indicators of patient outcome, with advanced age decreasing the probability of AICU admission.

### Mechanical ventilation

**Figure 4:**
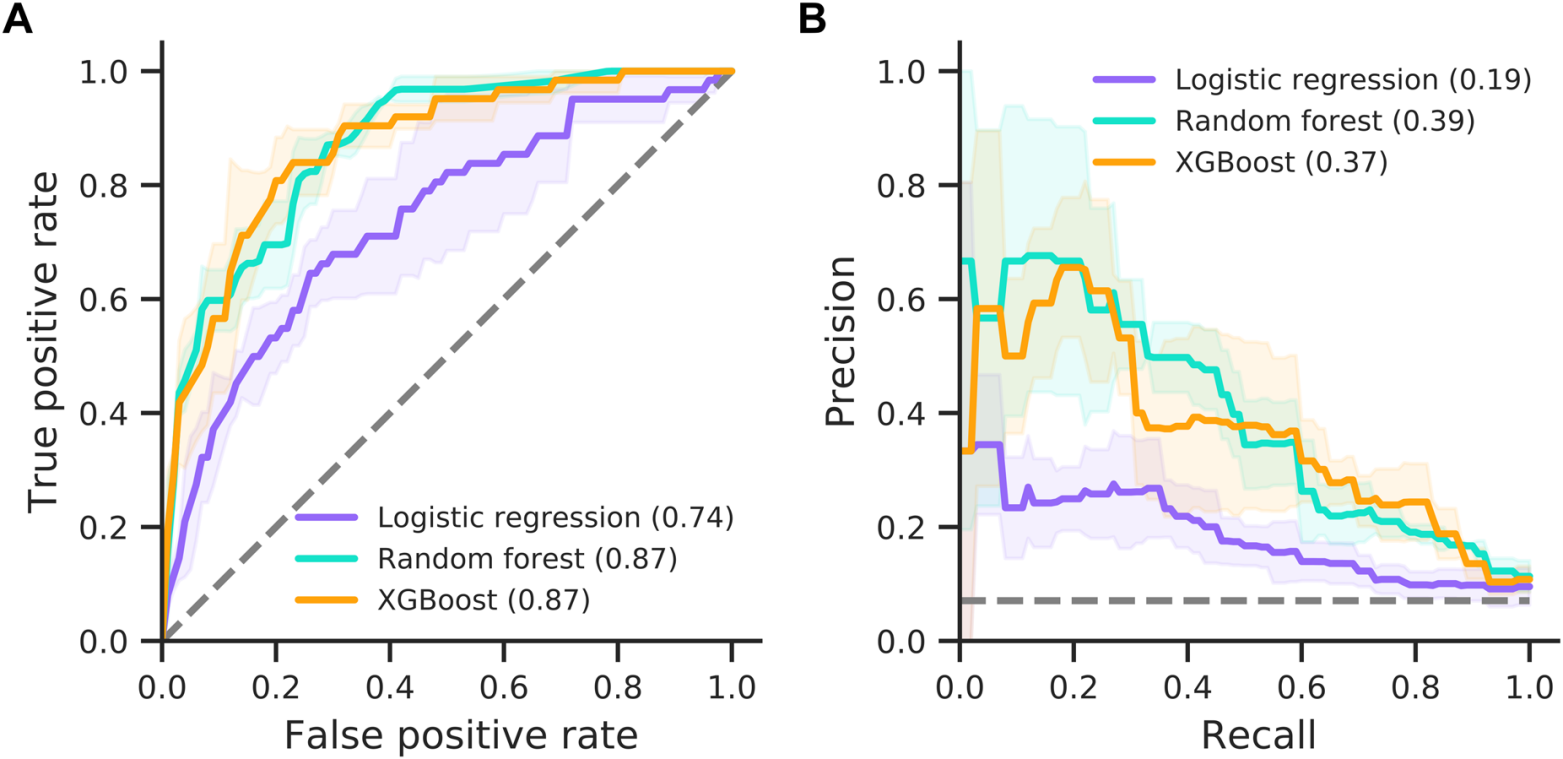
Prediction performance for mechanical ventilation. Model performances for the logistic regression, random forest and XGBoost models are shown as ROC (**A**) and precision-recall curves (**B**). AUC is provided in brackets. Solid lines and shaded areas indicate the mean and standard deviation across three cross-validation folds, respectively. Dashed lines indicate random classifiers.

For mechanical ventilation prediction, we categorised patients into those that needed a ventilator (e.g., patients receiving SIMV, BIPAP or APRV ventilation) and control patients that either were able to breathe normally or required minimal assistance (e.g., those patients receiving oxygen via nasal cannulae or face masks). Prediction performance on this endpoint is comparable to prediction of AICU admission (Fig. 4). Specifically, random forest and XGBoost perform best, reaching AUCs of 0.87, while logistic regression reaches 0.74 (Table 4). This result is expected since most patients receive mechanical ventilation in AICU, meaning the ventilation cohort is a subset of the critical care cohort (55 of 62 target patients in Cohort B are target patients in Cohort A). Notably, all models show a decrease in F1 scores and stability when predicting this clinical endpoint (Table 4 and Fig. 4). This is most likely due to a higher class-imbalance and lower number of patients receiving ventilation. However, both the random forest and XGBoost models maintain good calibration of predicted risk against true outcomes with Brier scores of 0.06 (Supplementary Fig. S3B).

**Figure 5:**
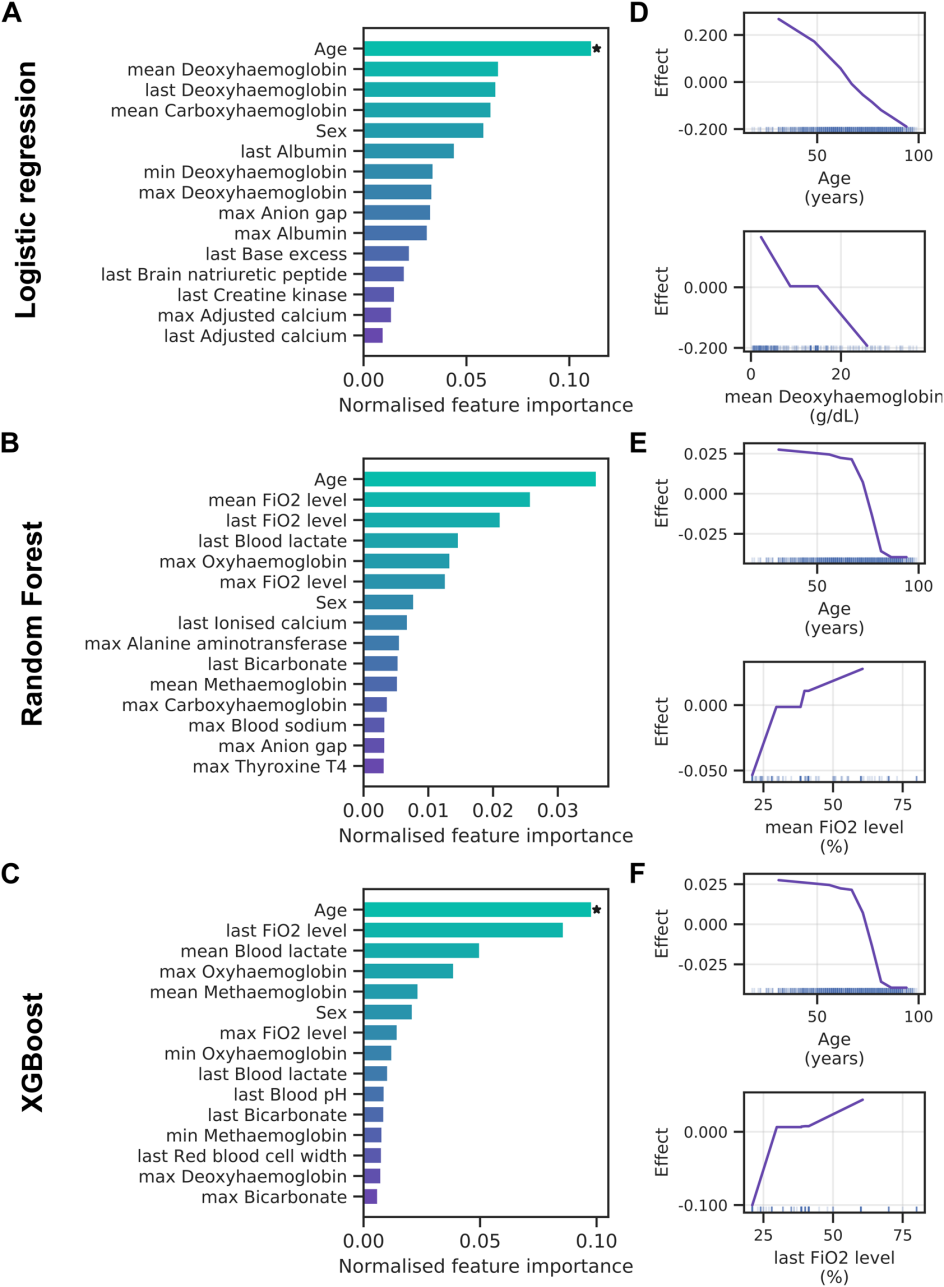
Feature importance for mechanical ventilation. Permutation feature importance for the random forest (**A**), logistic regression (**B**) and XGBoost (**C**) models. Only the top 15 features are shown. Asterisks mark features with importance scores significantly different from zero across three cross-validation folds with t-test *p* value thresholds of 5% ( ∗) and 1% (∗ ∗). (**D–F**) Accumulated local effects plots for the logistic regression (**D**), random forest (**E**) and XGBoost models (**F**). The top two features according to permutation feature importance are shown for each model. Vertical bars at the bottom indicate feature values observed in the data set.

Feature importance analysis for the logistic regression shows a large effect of patient age and deoxyhaemoglobin levels (Fig. 5A). This mirrors the results for AICU admission. Both tree-based methods rank patient age as well as the fraction of inspired oxygen (FiO_2_) and blood lactate levels highly (Fig. 5B,C), although few contributions are significant. In general, all models rely on a broader set of features for the ventilation endpoint. ALE analysis shows younger patients had an increased probability of receiving ventilation (Fig. 5D–F), which agrees with an inherent bias towards younger age when comparing ventilated with non-ventilated patients (Supplementary Fig. S5B). In addition, a low deoxyhaemoglobin level and a high fraction of inspired oxygen were associated with a poor prognosis.

Taken together, models show good performance when predicting ventilation, albeit with a decreased model stability (higher standard deviation) and F1 scores. Patient age and oxygenation status are most predictive of poor outcome, with additional contributions from blood test values, such as lactate and deoxyhaemoglobin levels.

### Mortality

**Figure 6:**
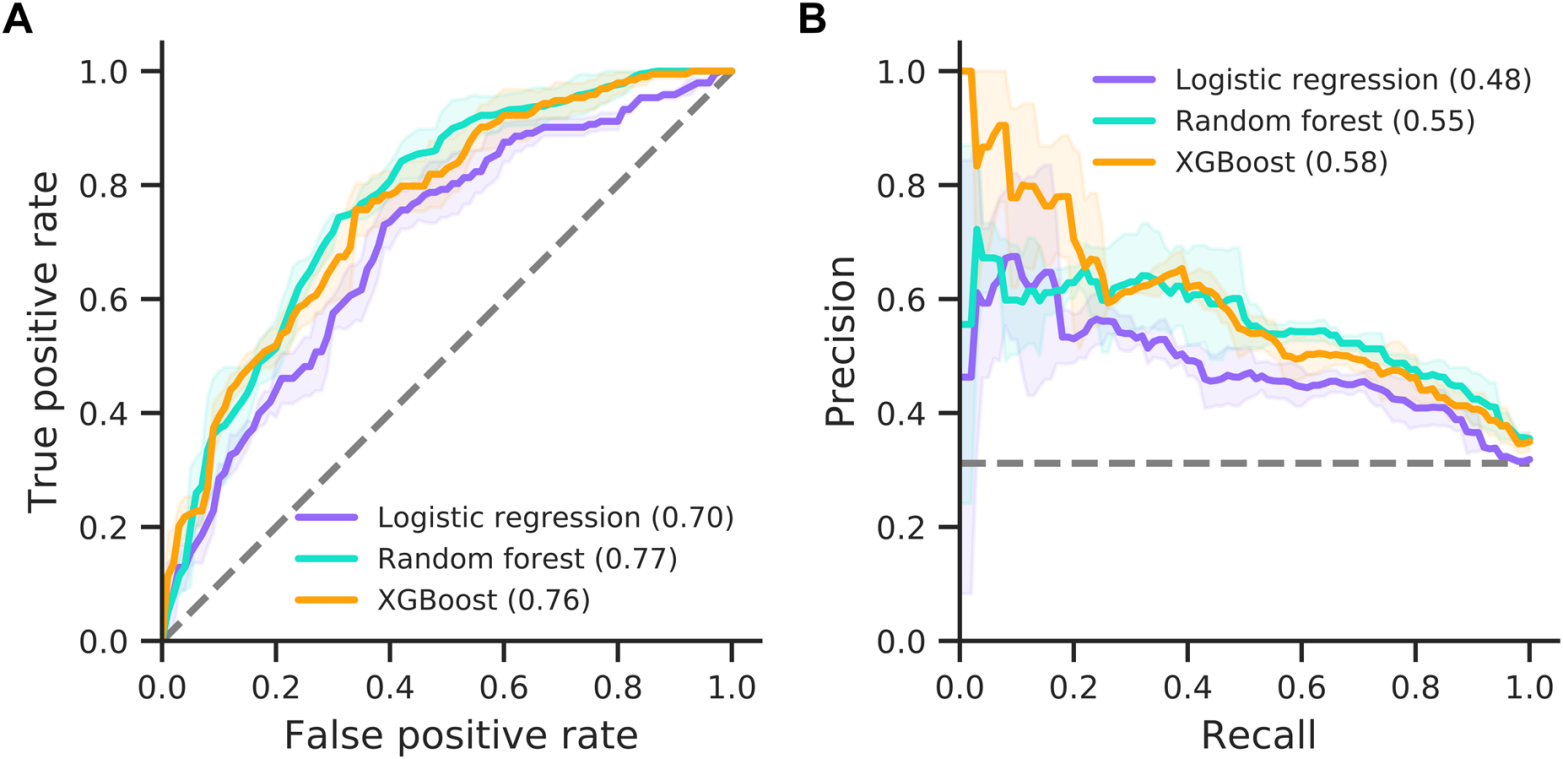
Prediction performance for mortality. Model performances for the logistic regression, random forest and XGBoost models are shown as ROC (**A**) and precision-recall curves (**B**). AUC is provided in brackets. Solid lines and shaded areas indicate the mean and standard deviation across three cross-validation folds, respectively. Dashed lines indicate random classifiers.

The performance of all three models shows a marked decrease in AUC-ROC when predicting mortality (Fig. 6). The logistic regression reaches an AUC of 0.70, whereas random forest and XGBoost reach 0.77 and 0.76, respectively. However, all models show improved precision with F1 scores of 0.56–0.61, reaching their highest values among all clinical endpoints (Table 4).

**Figure 7:**
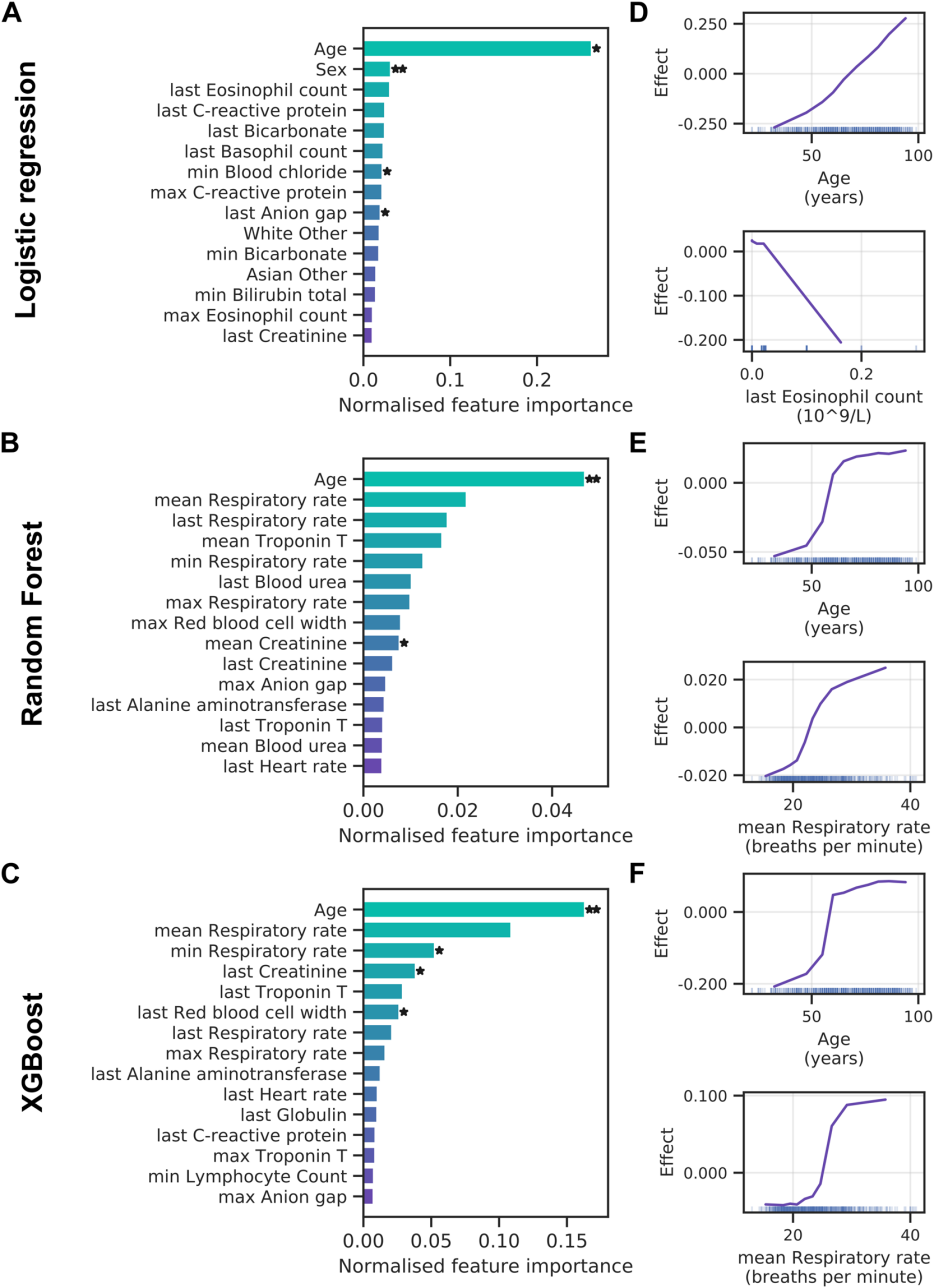
Feature importance for mortality. (**A–C**) Permutation feature importance for the logistic regression (**A**), random forest (**B**) and XGBoost (**C**) models. Only the top 15 features are shown. Asterisks mark features with importance scores significantly different from zero across three cross-validation folds with t-test *p* value thresholds of 5% ( ∗) and 1% (∗ ∗). (**D–F**) Accumulated local effects plots for the logistic regression (**D**), random forest (**E**) and XGBoost models (**F**). The top two features according to permutation feature importance are shown for each model. Vertical bars at the bottom indicate feature values observed in the data set.

Predictions from the logistic regression model are dominated by patient age, with patient sex adding a small but significant contribution (Fig. 7A). Similarly, tree-based methods rely heavily on age for their predictions, with smaller contributions of respiratory rate, Troponin T and creatinine levels (Fig. 7B,C). More generally, prediction of mortality shows several blood tests that are not strictly related to oxygenation status among the important features. ALE analysis shows that advanced age is predictive of higher mortality (Fig. 7D–F). This agrees with a bias towards older age in patients that die in hospital (Supplementary Fig. S5C). Moreover, low eosinophil counts and high respiratory rates increase the risk of mortality in our models (Fig. 7D–F).

In summary, our models show an increased F1 score but lower AUC-ROC performance when predicting mortality. Feature importance scores reveal a high and significant contribution of patient age with advanced age contributing to poor patient outcomes.

## Discussion

Disease severity can vary dramatically between COVID-19 patients, ranging from asymptomatic infection to severe respiratory distress and failure. To evaluate the potential of an early stratification of hospitalised patients into risk groups, we built machine learning models from EHR care data of confirmed COVID-19 positive patients, aimed at predicting one of three clinical endpoints: admission to AICU, the need for mechanical ventilation and mortality. On all three cohorts, our models reach good performance with the best model showing AUC-ROC between 0.76 and 0.87. Overall, machine learning methods can thus reliably predict poor outcomes for COVID-19 patients from early clinical data, available during the ED stay of patients.

The most predictive feature for all three endpoints was patient age, followed by indicators of patients’ oxygenation status, including fraction of inspired oxygen and respiratory rate. Given that SARS-CoV-2 causes an infection of the respiratory tract, which can lead to severe respiratory distress, these results were to be expected. Our findings are supported by similar works, in which age is consistently found to be the most important feature^14–16^. However, we note that other potential indicators for severe viral infection, like increased temperature and markers of immune system activation, e.g. C-reactive protein, are less prominent in our feature importance scores. Overall, prediction of mortality relies more strongly on blood tests as opposed to indicators of oxygen supply observed in other endpoints. The reason for this observation and its clinical significance merits further investigation. Our ALE analysis reveals that lower patient age contributes to an increased probability of receiving mechanical ventilation and critical care in AICU, while coinciding with lower mortality. We also note that Docherty et al*.* find that 17% of COVID-19 patients require admission to a High Dependency or Intensive Care Unit^29^, which is similar to 15% of patients in our data. Conversely, our findings regarding the importance of features relating to patients’ oxygenation status are not corroborated by other works. Specifically, other studies find that one important predictor of patient outcome is the level of lactate dehydrogenase^15,16^, which, although present in our data set, does not significantly contribute to predictions.

A novel aspect of the present analysis is the use of data limited to a patient's first few hours in ED. While this perhaps more accurately reflects the data available at the time of admission, it may well come at the cost of missing important information, such as medical history or primary care data, for predicting patient outcome. This may explain the comparative difficulty in predicting mortality, since a patient's overall chance of surviving infection may depend heavily on their medical history. Also note that, in our analysis, all patients were considered together for mortality prediction and the cohort was not further split according to confounding factors such as age or sex. In addition, mortality data for recent hospital admissions are by their nature censored, with clinical endpoints for patients who remain in hospital not yet fully known.

While we base our study on a comparatively large data set from a two-site NHS hospital trust, longitudinal information from additional treatment centres and geographic regions may improve a model’s ability to generalise. We note that such data is currently unavailable for COVID-19. However, future studies may benefit from a multicentre approach. As a result of limited data and the imbalanced cohorts, model stability remains a major challenge. While we use minority class oversampling, inverse class weights and stratified threefold cross validation to mitigate this issue, large uncertainties in model results persist, and many predictions do not reach statistical significance. Increased patient numbers, in particular among target patients, may lead to more conclusive results. Once such data is available, more complex models, such as deep neural networks, may achieve higher prediction performance. A key aspect which should be considered in such works is the prediction horizon, which impacts on how useful a model could be.

In conclusion, our models represent a first step towards the prediction of COVID-19 patient pathways in hospital at the point of admission in the emergency department. While they succeed in predicting patient outcomes and reveal critical clinical variables that may influence patient trajectories, larger data sets and further analyses are required to draw clinically relevant conclusions.

## Supplementary Information

Supplementary Information.

## References

[1] Wu Z, McGoogan JM (2020). Characteristics of and important lessons from the Coronavirus Disease 2019 (COVID-19) outbreak in China: summary of a report of 72 314 cases from the Chinese Center for Disease Control and Prevention. JAMA.

[2] Yang X (2020). Clinical course and outcomes of critically ill patients with SARS-CoV-2 pneumonia in Wuhan, China: a single-centered, retrospective, observational study. Lancet Respir. Med..

[3] Klok FA (2020). Incidence of thrombotic complications in critically ill ICU patients with COVID-19. Thromb. Res..

[4] Anderson RM, Heesterbeek H, Klinkenberg D, Hollingsworth TD (2020). How will country-based mitigation measures influence the course of the COVID-19 epidemic?. Lancet.

[5] Vizcaychipi MP (2020). Early detection of severe COVID-19 disease patterns define near real-time personalised care, bioseverity in males, and decelerating mortality rates. medRxiv.

[6] Novel Coronavirus Pneumonia Emergency Response Epidemiology Team (2020). The epidemiological characteristics of an outbreak of 2019 novel coronavirus diseases (COVID-19) in China. Chin. Cent. Dis. Control Prev..

[7] Chen T (2020). Clinical characteristics of 113 deceased patients with coronavirus disease 2019: retrospective study. BMJ.

[8] Goldstein BA, Navar AM, Pencina MJ, Ioannidis JPA (2017). Opportunities and challenges in developing risk prediction models with electronic health records data: a systematic review. J. Am. Med. Inform. Assoc..

[9] Wynants L (2020). Prediction models for diagnosis and prognosis of covid-19 infection: systematic review and critical appraisal. BMJ.

[10] Wang D (2020). Clinical characteristics of 138 hospitalized patients with 2019 novel coronavirus-infected pneumonia in Wuhan, China. JAMA.

[11] Arentz M (2020). Characteristics and outcomes of 21 critically ill patients with COVID-19 in Washington State. JAMA.

[12] Hu, L. *et al.* Risk factors associated with clinical outcomes in 323 COVID-19 patients in Wuhan, China. *medRxiv* 2020.03.25.20037721 (2020).

[13] Jiang X (2020). Towards an artificial intelligence framework for data-driven prediction of coronavirus clinical severity. CMC-Comput. Mater. Contin..

[14] Zhou F (2020). Clinical course and risk factors for mortality of adult inpatients with COVID-19 in Wuhan, China: a retrospective cohort study. Lancet.

[15] Gong J (2020). A tool for early prediction of severe Coronavirus Disease 2019 (COVID-19): a multicenter study using the risk Nomogram in Wuhan and Guangdong, China. Clin. Infect. Dis..

[16] Xie, J. *et al.* Development and external validation of a prognostic multivariable model on admission for hospitalized patients with COVID-19. *medRxiv* 2020.03.28.20045997 (2020).

[17] Pourhomayoun, M. & Shakibi, M. Predicting mortality risk in patients with COVID-19 using artificial intelligence to help medical decision-making. *medRxiv* 2020.03.30.20047308 (2020). 10.1016/j.smhl.2020.100178 PMC7832156 33521226

[18] Yan, L. *et al.* Prediction of criticality in patients with severe Covid-19 infection using three clinical features: a machine learning-based prognostic model with clinical data in Wuhan. *medRxiv* 2020.02.27.20028027 (2020).

[19] Lemaître G, Nogueira F, Aridas CK (2017). Imbalanced-learn: a Python toolbox to tackle the curse of imbalanced datasets in machine learning. J. Mach. Learn. Res..

[20] Zou H, Hastie T (2005). Regularization and variable selection via the elastic net. J. R. Stat. Soc. Ser. B Stat. Methodol..

[21] Breiman L (2001). Random forests. Mach. Learn..

[22] Chen, T. & Guestrin, C. XGBoost: a scalable tree boosting system. in *Proceedings of the 22nd ACM SIGKDD International Conference on Knowledge Discovery and Data Mining* 785–794 (2016).

[23] Fisher A, Rudin C, Dominici F (2019). All models are wrong, but many are useful: learning a variable’s importance by studying an entire class of prediction models simultaneously. J. Mach. Learn. Res..

[24] Apley, D. W. & Zhu, J. Visualizing the effects of predictor variables in black box supervised learning models. *ArXiv161208468 Stat* (2019).

[25] Harris CR (2020). Array programming with NumPy. Nature.

[26] McKinney, W. Data structures for statistical computing in Python. in *Proc. 9th Python Sci. Conf.* 56–61 (2010).

[27] Pedregosa F (2011). Scikit-learn: machine learning in Python. J. Mach. Learn. Res..

[28] Hunter JD (2007). Matplotlib: a 2D graphics environment. Comput. Sci. Eng..

[29] Docherty, A. B. *et al.* Features of 16,749 hospitalised UK patients with COVID-19 using the ISARIC WHO Clinical Characterisation Protocol. *medRxiv* 2020.04.23.20076042 (2020).

