# Supplementary material for: Early risk assessment for COVID-19 patients from emergency department data using machine learning
Source: Sci Rep. 2021 Feb 18;11:4200. doi: 10.1038/s41598-021-83784-y (PMC7892838; doi:10.1038/s41598-021-83784-y)
Supplement: Supplementary file 1 — Supplementary Information. [file 41598_2021_83784_MOESM1_ESM.pdf]

Supplementary information for

Early risk assessment for COVID-19 patients from emergency department data using machine learning

Authors
Frank S. Heldt[1,*], Marcela P. Vizcaychipi[2,3], Sophie Peacock[1], Mattia Cinelli[1], Lachlan McLachlan[1], Fernando Andreotti[1], Stojan Jovanović[1], Robert Dürichen[1], Nadezda Lipunova[1], Robert A. Fletcher[1], Anne Hancock[1], Alex McCarthy[2], Richard A. Pointon[2], Alexander Brown[2], James Eaton[2], Roberto Liddi[1], Lucy Mackillop[1,4,5], Lionel Tarassenko[1,6], Rabia T. Khan[1]

Affiliations
[1]Sensyne Health plc, Schrodinger Building, Heatley Road, Oxford Science Park, Oxford, OX4 4GE.
[2]Chelsea and Westminster Hospital NHS Foundation Trust, 369 Fulham Road, London, SW10 9NH, UK.
[3]Academic Department of Anaesthesia & Intensive Care Medicine, Imperial College London, Chelsea & Westminster Campus, 369 Fulham Road, London, SW10 9NH, UK.
[4]Oxford University Hospitals NHS Foundation Trust, Women's Centre, John Radcliffe Hospital, Headley Way, Headington, Oxford, OX3 9DU, UK.
[5]Nuffield Department of Women's and Reproductive Health, University of Oxford, Women's Centre, John Radcliffe Hospital, Headley Way, Headington, Oxford, OX3 9DU, UK.
[6]Institute of Biomedical Engineering, Department of Engineering Science, University of Oxford, OX3 7DQ.
*Corresponding author

Supplemental figures

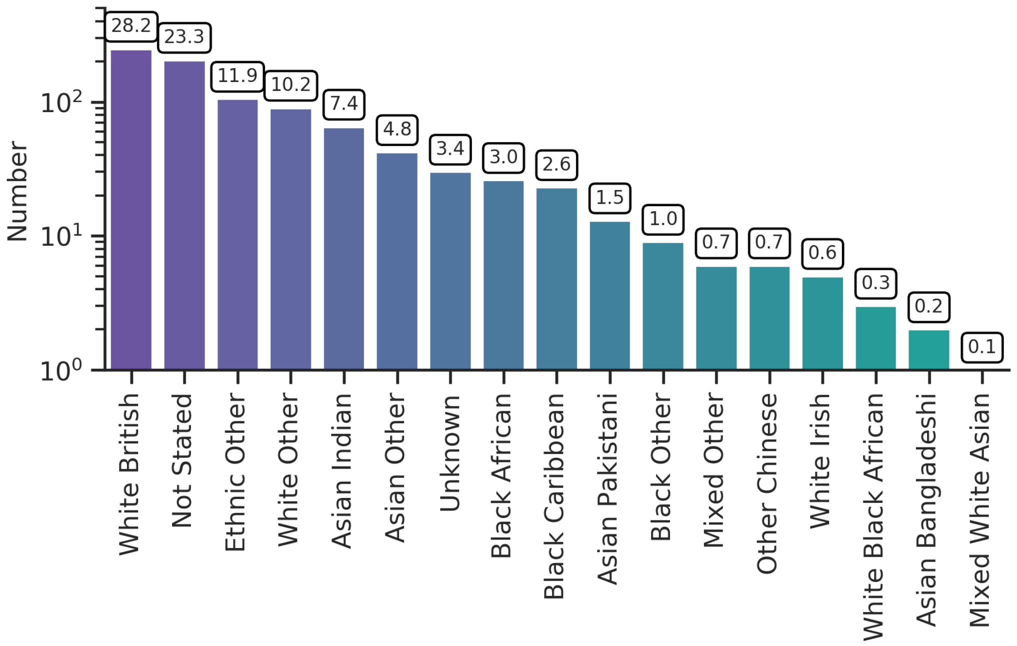

Supplementary Figure S1. Ethnicity distribution. *Number of patients in different ethnic groups. Numbers in squares indicate percentage over entire data set. Note logarithmic y-axis.*

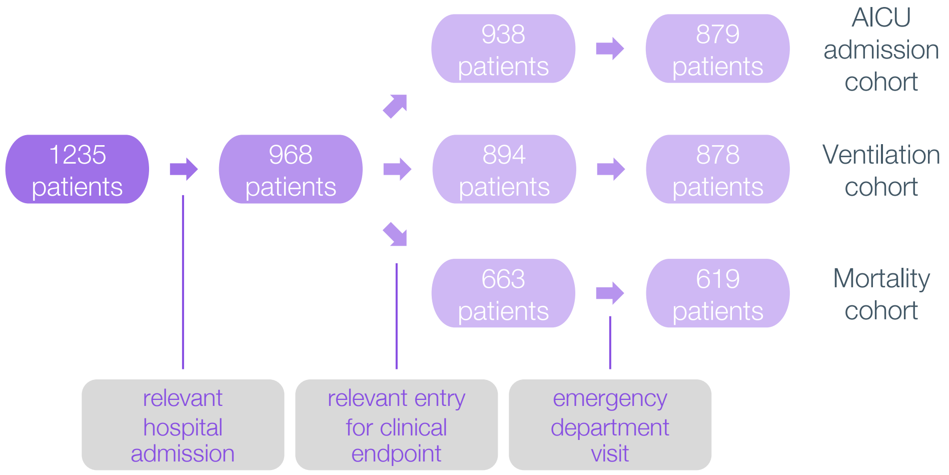

Supplementary Figure S2. Patient selection criteria. *1235 patients in total fell within the study parameters. A series of inclusion and exclusion criteria (see Methods for details) was applied to obtain a cohort for each of the three clinical endpoints.*

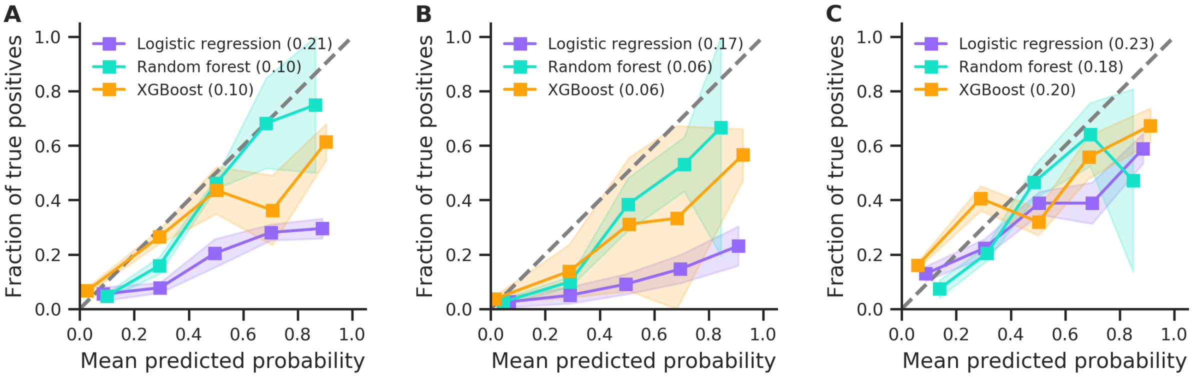

__Supplementary Figure S3. Model calibration.__ The fraction of true positive patients over the predicted risk is shown for AICU admission (A), ventilation (B) and mortality (C) prediction. Brier score is provided in brackets. Solid lines and shaded areas indicate the mean and standard deviation across three cross-validation folds, respectively. Dashed lines indicate perfect calibration.

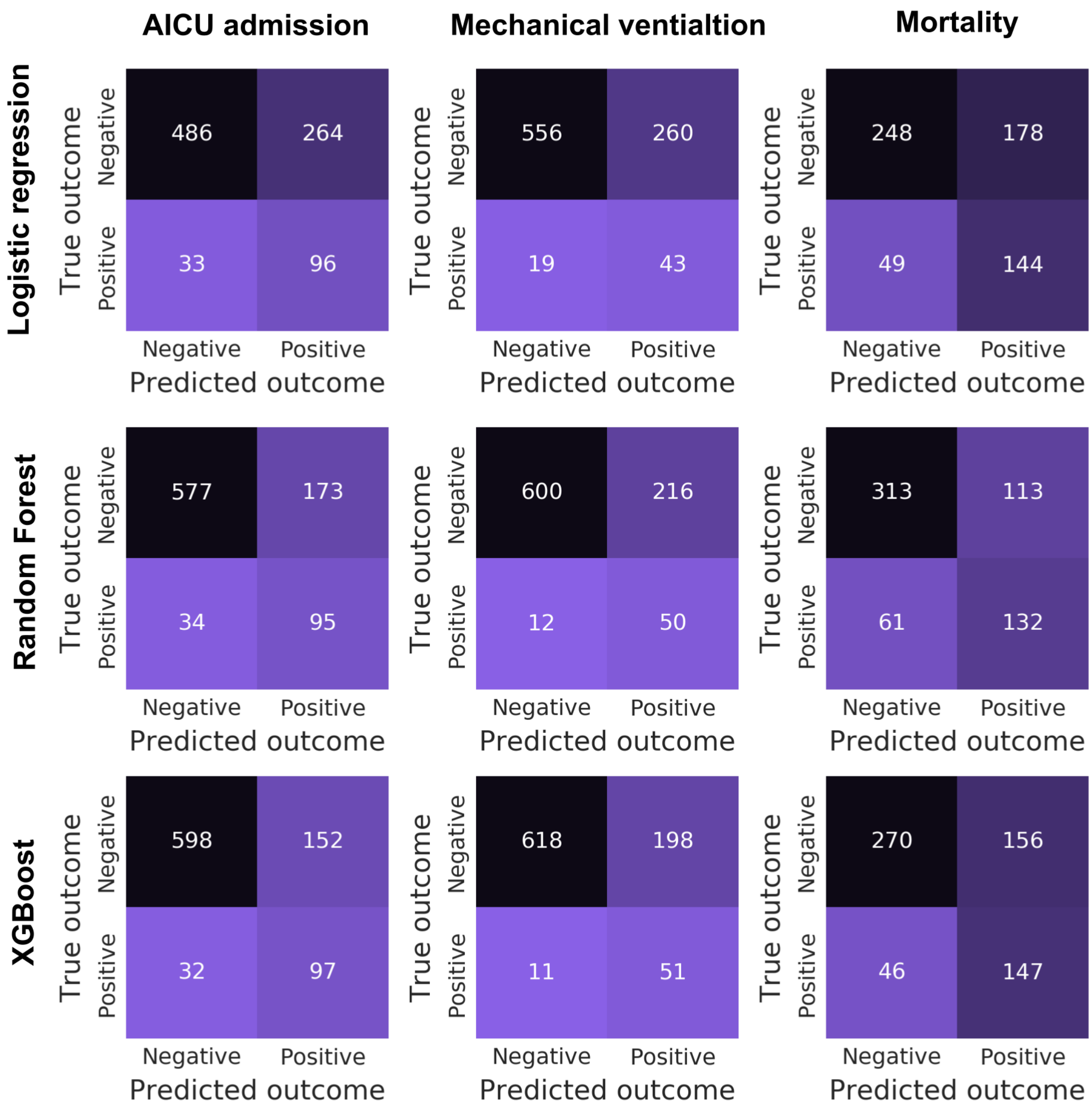

Supplementary Figure S4. Confusion matrices. *Confusion matrices of logistic regression, random forest and XGBoost (top to bottom) for predictions of AICU admission, mechanical ventilation and mortality (left to right). Patient numbers are shown in each field. Predictions are shown at each model's optimal classification threshold as determined by Youden's J statistic on the ROC curve.*

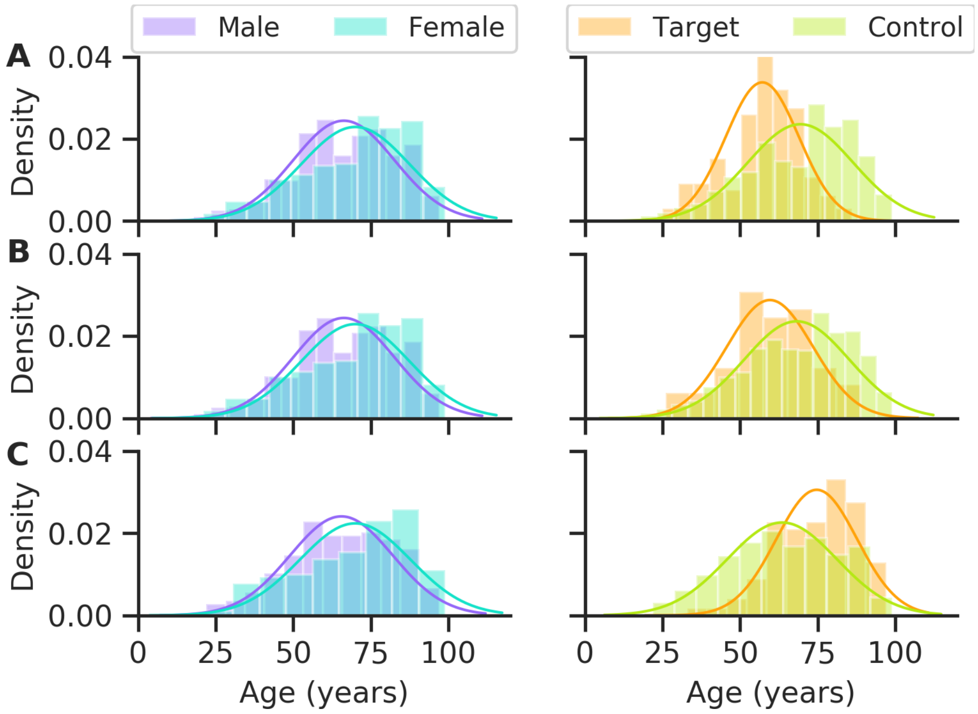

Supplementary Figure S5. Patient age distributions. *Distributions of patient age for AICU admission (A), ventilation (B) and mortality (C) cohorts are shown separated by patient sex (left) and target and control patients (right). Solid lines indicate fitted normal distributions.*

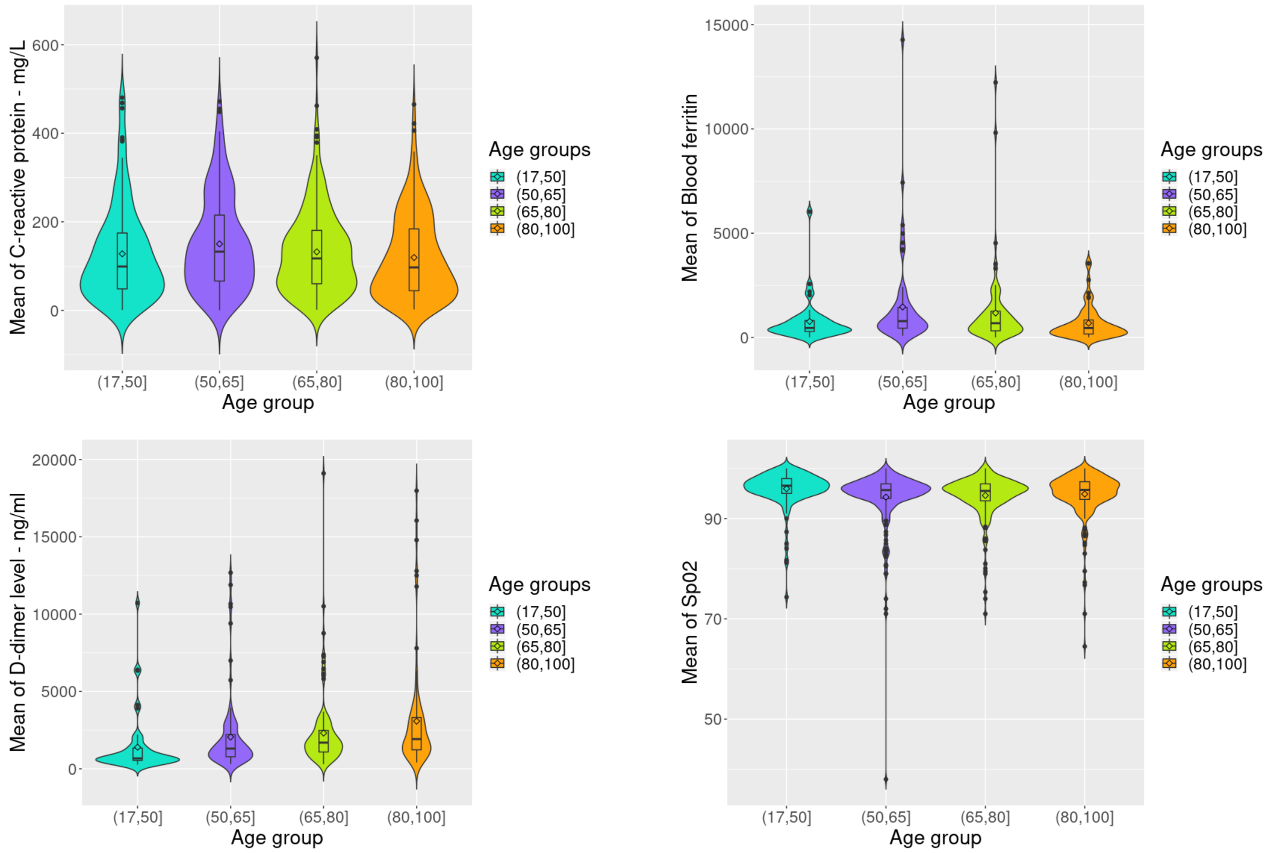

Supplementary Figure S6. Clinical indicators of disease severity by patient age. *Laboratory results for C-reactive protein, D-Dimer level, ferritin and peripheral oxygen saturation are shown in four age groups. Boxes indicate the median and inter-quartile range. Diamonds mark the mean.*

Supplemental Tables

Supplementary Table S1. *Composition of individual patient cohorts.*

	Cohort A (AICU admission)	Cohort B (Ventilation)	Cohort C (Mortality)
Patient age (years)			
Range	18.0 - 99.0	18.0 - 99.0	22.0 - 99.0
Overall mean (standard deviation)	67.6 (16.9)	67.6 (16.9)	67.0 (17.2)
Female mean (standard deviation)	70.0 (17.4)	70.0 (17.4)	69.9 (17.8)
Male mean (standard deviation)	66.3 (16.3)	66.3 (16.4)	65.5 (16.6)
Sex (number of patients)			
Female	324 (36.9%)	324 (36.9%)	213 (34.4%)
Male	554 (63.0%)	553 (63.0%)	405 (65.4%)
Unknown	1 (0.1%)	1 (0.1%)	1 (0.2%)
Ethnicity (number of patients)			
White British	248 (28.2%)	248 (28.2%)	170 (27.5%)
Not Stated	205 (23.3%)	205 (23.3%)	134 (21.6%)
Ethnic Other	105 (11.9%)	105 (12.0%)	72 (11.6%)
White Other	90 (10.2%)	90 (10.3%)	58 (9.4%)
Asian Indian	65 (7.4%)	65 (7.4%)	53 (8.6%)
Asian Other	42 (4.8%)	42 (4.8%)	34 (5.5%)
Unknown	30 (3.4%)	30 (3.4%)	28 (4.5%)
Black African	26 (3.0%)	25 (2.8%)	21 (3.4%)
Black Caribbean	23 (2.6%)	23 (2.6%)	16 (2.6%)
Asian Pakistani	13 (1.5%)	13 (1.5%)	8 (1.3%)
Other	32 (3.6%)	32 (3.6%)	25 (4.0%)

Supplementary Table S2. *Clinical features and their coverage in the data set.*

Feature (unit)	Coverage (%)
**Demographics**	
Age (years)	100.0
Sex	99.9
Ethnicity	73.3
**Vitals**	
Heart rate (beats per minute)	99.3
Respiratory rate (breaths per minute)	99.3
Temperature (degrees Celcius)	99.1
FiO2 level (%)	26.8
**Laboratory measurements**	
Haemoglobin (g/L)	90.6
Red blood cell width (%)	90.2
Haematocrit (L/L)	90.2
Mean corpuscular Haemoglobin (pg)	90.2
Mean corpuscular volume (fL)	90.2
Monocyte count (10^9/L)	90.2
Eosinophil count (10^9/L)	90.2
Neutrophil count (10^9/L)	90.2
Lymphocyte Count (10^9/L)	90.2
Red blood cell count (10^9/L)	90.2
MCHC (g/L)	90.2
Basophil count (10^9/L)	90.2
White blood cells (10^9/L)	90.2
Nucleated red blood cell count (10^9/L)	90.1
Mean platelet volume (fL)	90.0
Platelet count (10^9/L)	89.9
Blood urea (mmol/L)	89.2
Blood chloride (mmol/L)	89.2
Creatinine (µmol/L)	89.1
Blood sodium (mmol/L)	88.9
Alkaline phosphatase (U/L)	86.3
C-reactive protein (mg/L)	85.0
Albumin (g/dL)	84.9
Bilirubin total (µmol/L)	83.2
Blood potassium (mmol/L)	83.2
Alanine aminotransferase (U/L)	81.8
Troponin T (ng/L)	59.8
Prothrombin time (seconds)	53.7
Act. partial thromboplastin time (seconds)	53.7
Fibrinogen (g/L)	52.9
Blood calcium (mmol/L)	52.9
Adjusted calcium (mmol/L)	52.3
Blood phosphate (mmol/L)	52.0
Blood total protein (g/L)	49.7
Globulin (g/L)	49.0
Creatine kinase (U/L)	42.5
Glucose (mmol/L)	40.5
D-Dimer level (ng/ml)	38.0

Blood LDH level (U/L)	35.6
Blood lactate (mmol/L)	27.3
Blood ferritin (ng/ml)	27.1
Bicarbonate (mmol/L)	27.0
Oxygen partial pressure (kPa)	26.8
Blood pH	26.8
Ionised calcium (mmol/L)	26.8
Anion gap (mmol/L)	26.8
Methaemoglobin (%)	26.7
Oxyhaemoglobin (g/dL)	26.7
Deoxyhaemoglobin (g/dL)	26.6
Carboxyhaemoglobin (%)	26.6
Blood magnesium (mmol/L)	20.4
Amylase (U/L)	18.1
Base excess (mmol/L)	11.8
Thyroxine T4 (pmol/L)	10.7
Thyroid stimulating hormone (mU/L)	10.4
Brain natriuretic peptide (pg/ml)	9.2
Cortisol (nmol/L)	8.1

Supplementary Table S3. Clinical features for control and target patients in the AICU admission cohort.

Feature	Control patients			Targe patients		
	Mean	Std. dev.	n patients	Mean	Std. dev.	n patients
Age	69.43	16.939	750	57.14	11.862	129
Sex female	38.5%		289	27.1%		35
Sex male	61.3%		460	72.9%		94
Sex unknown	0.1%		1	0.0%		0
Act. partial thromboplastin time	32.74	7.144	396	32.72	4.471	76
Adjusted calcium	2.29	0.14	401	2.25	0.113	59
Alanine aminotransferase	47.31	128.759	614	68.56	77.346	105
Albumin	34.35	4.811	640	33.52	5.012	106
Alkaline phosphatase	99.53	69.465	650	99.2	76.301	109
Amylase	64.27	64.411	137	200.68	391.406	22
Anion gap	14.15	4.059	188	14.93	4.167	48
Base excess	3.07	3.235	86	3.2	2.564	18
Basophil count	0.01	0.039	678	0.01	0.05	115
Bicarbonate	23.17	4.725	189	22.12	4.532	48
Bilirubin total	13.04	9.176	627	15.32	16.118	104
Blood calcium	2.19	0.159	406	2.14	0.138	59
Blood chloride	100.3	6.853	669	97.83	5.805	115
Blood ferritin	1001.79	1517.547	199	1355.79	2248.35	39
Blood lactate	1.8	1.758	191	1.58	1.014	49
Blood LDH level	487.92	520.88	261	644.69	261.974	52
Blood magnesium	0.82	0.15	149	0.93	0.283	30
Blood pH	7.43	0.073	188	7.44	0.085	48
Blood phosphate	1.05	0.416	397	1.07	0.485	60
Blood potassium	4.12	0.604	622	4.15	0.724	109
Blood sodium	136.03	6.984	666	133.6	5.499	115
Blood total protein	68.32	6.449	382	68.83	6.898	55
Blood urea	9.37	8.069	668	7.87	10.412	116
Brain natriuretic peptide	178.43	388.285	71	215.4	516.39	10
C-reactive protein	125.37	97.382	637	177.04	107.116	110
Carboxyhaemoglobin	2.57	1.601	187	2.04	1.267	47
Cortisol	754.45	385.858	55	804.06	443.062	16
Creatine kinase	495.96	1159.209	312	606.26	1049.317	62
Creatinine	126.77	128.798	670	106.21	82.578	113
D-Dimer level	2357.71	2761.83	291	1946.77	2368.467	43
Deoxyhaemoglobin	15.52	16.663	186	8.84	8.663	48
Eosinophil count	0.03	0.083	678	0.04	0.309	115
Fibrinogen	6.5	1.941	393	7.19	1.933	72
FiO2 level	35.5	22.231	188	54.8	26.867	48
Globulin	33.29	5.489	377	34.69	5.362	54
Glucose	8.7	5.952	290	9.22	6.285	66
Haematocrit	0.4	0.06	678	0.43	0.052	115
Haemoglobin	132.76	20.424	680	141.34	18.539	116
Heart rate	90.32	22.775	748	98.65	29.783	125
Ionised calcium	1.11	0.09	188	1.11	0.076	48
Lymphocyte Count	1.11	2.192	678	0.99	0.645	115
MCHC	332.72	10.608	678	332.39	12.287	115
Mean corpuscular Haemoglobin	29.79	2.512	678	29.17	3.078	115

Mean corpuscular volume	89.59	7.218	678	87.75	9.102	115
Mean platelet volume	8.48	1.128	676	8.74	1.169	115
Methaemoglobin	0.69	0.335	187	0.63	0.3	48
Monocyte count	0.62	0.429	678	0.57	0.399	115
Neutrophil count	6.79	4.27	678	8.28	5.968	115
Nucleated red blood cell count	0.01	0.072	677	0.04	0.257	115
Oxygen partial pressure	9.18	5.217	188	10.0	4.159	48
Oxyhaemoglobin	81.42	17.171	187	88.55	9.503	48
Platelet count	238.12	109.581	675	227.6	102.462	115
Prothrombin time	16.83	12.364	396	14.91	4.196	76
Red blood cell count	4.48	0.725	678	4.91	0.699	115
Red blood cell width	14.44	2.234	678	13.67	1.257	115
Respiratory rate	22.83	5.246	748	27.6	6.213	125
Sp02	95.25	3.894	748	92.11	7.223	125
Temperature	36.94	0.882	748	37.32	0.788	123
Thyroid stimulating hormone	1.4	1.073	72	1.26	0.816	19
Thyroxine T4	14.12	2.526	75	12.94	2.398	19
Troponin T	138.92	908.983	448	131.26	511.365	78
White blood cells	8.57	4.978	678	9.91	6.385	115

Supplementary Table S4. Clinical features for control and target patients in the mechanical ventilation cohort.

Feature	Control patients			Targe patients		
	Mean	Std. dev.	n patients	Mean	Std. dev.	n patients
Age	68.24	16.918	816	59.6	13.979	62
Sex female	37.9%		309	24.2%		15
Sex male	62.0%		506	75.8%		47
Sex unknown	0.1%		1	0.0%		0
Act. partial thromboplastin time	32.41	5.672	396	32.5	3.469	44
Adjusted calcium	2.29	0.14	383	2.2	0.105	21
Alanine aminotransferase	50.86	133.467	596	55.81	49.003	57
Albumin	34.35	4.818	623	31.37	4.163	54
Alkaline phosphatase	100.91	71.498	632	95.71	83.744	57
Amylase	87.42	173.341	137	76.0	33.067	8
Anion gap	14.27	4.173	189	14.66	3.811	47
Base excess	3.1	3.221	86	2.75	2.04	18
Basophil count	0.01	0.04	686	0.01	0.054	57
Bicarbonate	23.13	4.789	190	21.96	4.111	47
Bilirubin total	13.39	10.15	610	13.66	16.255	56
Blood calcium	2.19	0.158	389	2.09	0.1	22
Blood chloride	100.09	6.864	660	99.3	5.745	61
Blood ferritin	1029.87	1600.614	174	1584.07	2647.655	27
Blood lactate	1.79	1.762	192	1.66	1.177	48
Blood LDH level	514.71	544.185	239	654.12	291.713	25
Blood magnesium	0.84	0.19	144	0.78	0.076	7
Blood pH	7.43	0.075	189	7.45	0.077	47
Blood phosphate	1.05	0.421	382	1.0	0.367	21
Blood potassium	4.13	0.634	606	4.06	0.513	57
Blood sodium	135.85	6.955	656	133.84	4.947	59
Blood total protein	68.31	6.209	365	66.59	7.89	17
Blood urea	9.41	8.837	656	7.17	4.794	57
Brain natriuretic peptide	199.01	426.737	71	30.12	35.228	8
C-reactive protein	129.75	100.408	626	152.38	95.4	57
Carboxyhaemoglobin	2.55	1.595	188	2.11	1.319	46
Cortisol	748.79	380.339	57	834.21	466.747	14
Creatine kinase	533.49	1216.465	299	664.47	1369.27	36
Creatinine	127.71	131.88	658	102.99	49.676	58
D-Dimer level	2383.53	2764.831	260	1468.22	951.304	23
Deoxyhaemoglobin	15.44	16.709	187	9.07	8.309	47
Eosinophil count	0.03	0.15	686	0.0	0.013	57
Fibrinogen	6.57	1.991	389	6.94	1.992	44
FiO2 level	35.32	22.014	189	55.46	26.1	47
Globulin	33.27	5.377	359	35.88	7.928	17
Glucose	8.75	5.091	307	7.84	3.754	48
Haematocrit	0.4	0.06	686	0.43	0.051	57
Haemoglobin	133.46	20.556	688	139.69	17.431	61
Heart rate	91.46	24.533	812	92.41	16.993	62
Ionised calcium	1.11	0.09	189	1.1	0.079	47
Lymphocyte Count	1.12	2.188	686	0.93	0.46	57
MCHC	332.71	10.725	686	329.18	12.752	57
Mean corpuscular Haemoglobin	29.74	2.55	686	29.49	2.823	57

Mean corpuscular volume	89.41	7.314	686	89.66	9.022	57
Mean platelet volume	8.49	1.131	684	8.59	1.15	57
Methaemoglobin	0.69	0.341	188	0.61	0.265	47
Monocyte count	0.62	0.436	686	0.53	0.394	57
Neutrophil count	7.0	4.504	686	6.91	4.887	57
Nucleated red blood cell count	0.01	0.074	685	0.06	0.358	57
Oxygen partial pressure	9.25	5.223	189	9.73	4.204	47
Oxyhaemoglobin	81.52	17.236	188	88.24	9.093	47
Platelet count	237.27	110.316	683	220.56	99.242	57
Prothrombin time	16.45	10.887	396	14.12	2.294	44
Red blood cell count	4.52	0.741	686	4.8	0.641	57
Red blood cell width	14.41	2.197	686	13.36	1.243	57
Respiratory rate	23.27	5.527	812	27.42	6.921	62
Sp02	94.97	4.548	812	92.89	4.736	62
Temperature	36.97	0.879	811	37.37	0.822	62
Thyroid stimulating hormone	1.31	1.009	71	1.3	0.827	17
Thyroxine T4	13.97	2.55	74	13.12	2.404	17
Troponin T	135.09	908.292	430	38.45	116.419	42
White blood cells	8.79	5.224	686	8.4	5.115	57

Supplementary Table S5. Clinical features for control and target patients in the mortality cohort.

Feature	Control patients			Targe patients		
	Mean	Std. dev.	n patients	Mean	Std. dev.	n patients
Age	63.51	17.659	426	74.68	13.08	193
Sex female	37.1%		158	28.5%		55
Sex male	62.7%		267	71.5%		138
Sex unknown	0.2%		1	0.0%		0
Act. partial thromboplastin time	32.5	7.545	215	34.2	6.191	98
Adjusted calcium	2.28	0.137	231	2.3	0.143	122
Alanine aminotransferase	44.9	67.58	347	53.01	159.456	149
Albumin	35.24	4.665	356	33.66	4.241	160
Alkaline phosphatase	95.22	71.537	362	99.55	62.477	161
Amylase	61.33	41.903	75	96.77	197.49	39
Anion gap	13.51	3.475	90	15.99	3.45	35
Base excess	3.09	3.34	47	1.84	1.278	8
Basophil count	0.01	0.043	380	0.01	0.034	171
Bicarbonate	23.75	4.356	91	20.55	3.719	35
Bilirubin total	12.97	9.983	349	14.17	10.571	154
Blood calcium	2.19	0.156	232	2.18	0.161	123
Blood chloride	99.51	5.879	372	100.6	8.141	168
Blood ferritin	1123.89	1796.91	96	804.73	865.972	49
Blood lactate	1.49	0.892	92	1.98	1.285	36
Blood LDH level	488.29	339.953	144	483.72	218.632	82
Blood magnesium	0.82	0.153	91	0.87	0.166	53
Blood pH	7.44	0.065	90	7.42	0.079	35
Blood phosphate	1.0	0.386	228	1.1	0.428	119
Blood potassium	4.06	0.571	352	4.2	0.62	156
Blood sodium	135.21	5.842	372	136.45	8.435	167
Blood total protein	68.65	6.119	223	68.54	6.755	112
Blood urea	7.57	7.254	376	11.61	7.745	167
Brain natriuretic peptide	137.96	313.716	28	438.69	682.753	13
C-reactive protein	122.03	94.93	348	163.96	100.521	166
Carboxyhaemoglobin	2.55	1.555	90	2.24	1.299	35
Cortisol	726.0	349.839	26	1010.1	591.495	10
Creatine kinase	503.98	1087.446	158	589.26	1086.771	90
Creatinine	109.03	117.73	374	148.58	128.63	167
D-Dimer level	2091.32	2822.06	148	2851.9	2995.106	84
Deoxyhaemoglobin	14.14	16.575	90	13.47	15.584	34
Eosinophil count	0.03	0.093	380	0.01	0.037	171
Fibrinogen	6.53	1.905	212	7.01	2.084	94
FiO2 level	32.71	18.711	90	54.11	28.938	35
Globulin	32.98	5.298	219	34.1	5.689	110
Glucose	9.16	7.52	148	9.28	4.333	73
Haematocrit	0.41	0.056	380	0.4	0.067	171
Haemoglobin	135.5	19.39	380	132.91	22.485	172
Heart rate	91.52	25.893	424	96.07	27.05	192
Ionised calcium	1.1	0.081	90	1.12	0.105	35
Lymphocyte Count	1.04	0.591	380	1.21	4.229	171
MCHC	334.56	10.526	380	332.51	11.078	171
Mean corpuscular Haemoglobin	29.6	2.481	380	29.75	2.9	171

Mean corpuscular volume	88.51	7.137	380	89.48	8.217	171
Mean platelet volume	8.5	1.065	379	8.75	1.148	171
Methaemoglobin	0.7	0.306	90	0.71	0.312	35
Monocyte count	0.6	0.393	380	0.6	0.407	171
Neutrophil count	6.7	4.193	380	7.67	5.078	171
Nucleated red blood cell count	0.01	0.056	379	0.01	0.045	171
Oxygen partial pressure	8.81	4.285	90	11.28	6.31	35
Oxyhaemoglobin	82.63	17.161	90	84.54	15.853	35
Platelet count	241.96	111.173	379	228.48	96.361	170
Prothrombin time	16.5	13.515	215	16.99	7.062	98
Red blood cell count	4.6	0.686	380	4.51	0.833	171
Red blood cell width	14.09	2.0	380	14.95	2.293	171
Respiratory rate	22.69	4.997	424	25.97	6.463	192
Sp02	95.4	4.548	424	93.64	4.706	192
Temperature	37.07	0.803	423	36.9	0.964	191
Thyroid stimulating hormone	1.58	1.214	35	1.27	1.128	12
Thyroxine T4	13.52	2.555	35	13.57	2.21	13
Troponin T	42.01	121.515	226	293.37	1545.349	139
White blood cells	8.4	4.479	380	9.5	6.645	171

Supplementary Table S6. *Sensitivity and specificity on clinical endpoints (standard deviation shown in brackets) at each model's ideal classification threshold as determined by Youden's J statistic on the ROC curve.*

Model	Endpoint A (AICU admission)		Endpoint B (ventilation)		Endpoint C (mortality)	
	Sensitivity	Specificity	Sensitivity	Sensitivity	Sensitivity	Sensitivity
Logistic regression	0.72 (0.141)	0.68 (0.086)	0.73 (0.215)	0.66 (0.113)	0.74 (0.041)	0.59 (0.011)
Random forest	**0.75** (0.075)	0.77 (0.062)	**0.82** (0.074)	0.73 (0.077)	**0.76** (0.102)	**0.67** (0.072)
XGBoost	0.74 (0.062)	**0.80** (0.024)	0.76 (0.080)	**0.85** (0.057)	0.74 (0.056)	0.66 (0.004)
